# Supplementary material for: A national survey of videolaryngoscopes and alternative intubation devices in Hungary
Source: PLoS One. 2019 Oct 10;14(10):e0223645. doi: 10.1371/journal.pone.0223645 (PMC6786552; doi:10.1371/journal.pone.0223645)
Supplement: S1 Appendix — (DOCX) [file pone.0223645.s001.docx]

**Questions regarding the survey**

**Consent for participation and publishing**

Yes

No

**Age in years**

**Gender**

Male

Female

**Professional experience (Single best answer!)**

Trainee with less than 2 years of experience

Trainee with 2-5 years of experience

Specialist with overall less than 10 years of experience

Specialist with overall 10-20 years of experience

Specialist with more than 20 years of experience overall

**Which one of the followings is the most specific to your daily professional activity? (Single best answer!)**

Patient care, mainly anesthesia

Patient care, mainly intensive therapy

Patient care, other

Education

Administration

Other, non-patient care-related activity

**Place of work (Single best answer!)**

City/community Hospital

County Hospital

University Hospital

Private Hospital

Other

**Teaching activity (Single best answer!)**

Involved regularly in education of trainees (at least once per week on average)

Involved occasionally in education of trainees (at least once per month on average)

Involved occasionally in education of trainees (less than once per month on average)

Not involved in education at all

**Is any kind of videolaryngoscope available at any workstation at your workplace? (Single best answer!)**

Yes

No

I don’t know

**At which workstation at your workplace do you have a videolaryngoscope immediately/readily available? (Option for multiple answers!)**

None

We have one, but I actually don’t know where…

Surgery

Obstetrics

Ear-Nose-Throat

Maxillofacial surgery

Thoracic surgery

Vascular surgery

Urology

Traumatology

Emergency department

Orthopedics

Pediatric surgery

Intensive care unit

Other

**At which workstation at your workplace do you have a videolaryngoscope available within 10 minutes? (Option for multiple answers!)**

None

We have one, but I actually don’t know where…

Surgery

Obstetrics

Ear-Nose-Throat

Maxillofacial surgery

Thoracic surgery

Vascular surgery

Urology

Traumatology

Emergency department

Orthopedics

Pediatric surgery

Intensive care unit

Other

**Have you ever heard about any of the following devices?** **(Option for multiple answers!)**

Airtraq (Prodol Meditec, Guecho, Spain)

AP Venner (Venner Medical GmbH, Danischenhagen, Germany)

Bonfils (Karl Storz, Slough, UK)

Bullard (Circon, ACMI, Stamford, CT, USA)

C-MAC (Karl Storz, Slough, UK)

C-MAC D-blade (Karl Storz, Slough, UK)

Coopdech (Daiken Medical, Osaka, Japan)

C-Trach (previously, Laryngeal mask company, Henley-on-Thames, UK)

GlideScope (Verathon UK, Amersham, UK)

King Vision VL (Ambu, St Ives, UK)

Levitan FPS (Clarus Medical, Minneapolis, MN, USA)

McGrath 5 (Aircraft Medical, Edinburgh, UK)

McGrath Mac (Aircraft Medical, Edinburgh, UK)

Pentax AWS (Pentax, Tokyo, Japan)

Shikani intubating stylet (Clarus Medical, Minneapolis, MN, USA)

Upsherscope (Mercury Medical, Clearwater, FL, USA)

Vividtrac (Vivid Medical, Palo Alto, USA)

Wuscope (Pentax Precision instruments, Orangeburg, NY, USA)

None of the above

Other

**Which of the following devices are available at your workplace? (Option for multiple answers!)**

No videolaryngoscope is available at my workplace

A videolaryngoscope is available, but I am not sure about the brand

Airtraq (Prodol Meditec, Guecho, Spain)

AP Venner (Venner Medical GmbH, Danischenhagen, Germany)

Bonfils (Karl Storz, Slough, UK)

Bullard (Circon, ACMI, Stamford, CT, USA)

C-MAC (Karl Storz, Slough, UK)

C-MAC D-blade (Karl Storz, Slough, UK)

Coopdech (Daiken Medical, Osaka, Japan)

C-Trach (previously, Laryngeal mask company, Henley-on-Thames, UK)

GlideScope (Verathon UK, Amersham, UK)

King Vision VL (Ambu, St Ives, UK)

Levitan FPS (Clarus Medical, Minneapolis, MN, USA)

McGrath 5 (Aircraft Medical, Edinburgh, UK)

McGrath Mac (Aircraft Medical, Edinburgh, UK)

Pentax AWS (Pentax, Tokyo, Japan)

Shikani intubating stylet (Clarus Medical, Minneapolis, MN, USA)

Upsherscope (Mercury Medical, Clearwater, FL, USA)

Vividtrac (Vivid Medical, Palo Alto, USA)

Wuscope (Pentax Precision instruments, Orangeburg, NY, USA)

Other

**Have you ever used any of the following devices? (Option for multiple answers!)**

Airtraq (Prodol Meditec, Guecho, Spain)

AP Venner (Venner Medical GmbH, Danischenhagen, Germany)

Bonfils (Karl Storz, Slough, UK)

Bullard (Circon, ACMI, Stamford, CT, USA)

C-MAC (Karl Storz, Slough, UK)

C-MAC D-blade (Karl Storz, Slough, UK)

Coopdech (Daiken Medical, Osaka, Japan)

C-Trach (previously, Laryngeal mask company, Henley-on-Thames, UK)

GlideScope (Verathon UK, Amersham, UK)

King Vision VL (Ambu, St Ives, UK)

Levitan FPS (Clarus Medical, Minneapolis, MN, USA)

McGrath 5 (Aircraft Medical, Edinburgh, UK)

McGrath Mac (Aircraft Medical, Edinburgh, UK)

Pentax AWS (Pentax, Tokyo, Japan)

Shikani intubating stylet (Clarus Medical, Minneapolis, MN, USA)

Upsherscope (Mercury Medical, Clearwater, FL, USA)

Vividtrac (Vivid Medical, Palo Alto, USA)

Wuscope (Pentax Precision instruments, Orangeburg, NY, USA)

None of the above

Other

**How often do you use a videolaryngoscope? (Single best answer!)**

Never

Every day

At least once in a week

At least once in a month

At least once in a year

Less than once in a year

**Which of the following are indications for videolaryngoscopy? (Option for multiple answers!)**

I never use a videolaryngoscope

“Routine” airway management

“Predicted” difficult airway

“Unexpected” difficult airway

Education

Other

**Which kind of education have you received regarding videolaryngoscopy? (Single best answer!)**

I never received any education regarding videolaryngoscopy, and I never use videolaryngoscopy

I never received any education regarding videolaryngoscopy, but I use videolaryngoscopy

Compulsory education on simulators

Compulsory education on patients

Voluntary education on simulators

Voluntary education on patients

**Which of the following do you consider as a clinical alternative to videolaryngoscopy? (Option for multiple answers!)**

Direct laryngoscopy

Fibroscopy

Laryngeal mask

Surgical airway

Other

I don’t know any alternative device/procedure

**What was the rationale behind choosing a specific videolaryngoscope at your workplace? (Option for multiple answers!)**

There is no videolaryngoscope at my workplace

Actually, I don’t know

Price

Based on a short clinical trial

Based on scientific publications

Decision of the leader of the department

Based on the opinion of a local “airway experts”

Decision of the management without professional advice

**What is your overall opinion on videolaryngoscopes? (Single best answer!)**

I don’t have enough experience to draw a conclusion

Useless gadgets without real clinical benefits

Useful devices that should be used in all cases of advanced airway management

Useful devices that are beneficial only under specific circumstances

**Which kind of problems can be managed successfully with videolaryngoscopy according to you? (Option for multiple answers!)**

Patient cannot open his/her mouth at all

Difficulties visualizing the vocal cords appropriately

Difficulties with endotracheal tube placement although the vocal cords are fully visible

Airway contaminated with blood, secretions or stomach contents

Laryngeal edema

Suspected or definitive cervical spine injury

None of the above
