# Supplementary material for: A national survey of videolaryngoscopes and alternative intubation devices in Hungary
Source: PLoS One. 2019 Oct 10;14(10):e0223645. doi: 10.1371/journal.pone.0223645 (PMC6786552; doi:10.1371/journal.pone.0223645)
Supplement: S2 Appendix — (DOCX) [file pone.0223645.s002.docx]

**A kérdőív kérdései**

**Beleegyezik a kutatásban való részvételbe és az eredmények közzétételébe?**

Igen

Nem

**Életkor**

**Nem**

Férfi

Nő

**Szakmai tapasztalat (Egy válasz jelölhető!)**

Szakorvosjelölt kevesebb mint 2 év gyakorlattal

Szakorvosjelölt 2-5 év gyakorlattal

Szakorvos 10 évnél rövidebb teljes szakmai gyakorlattal

Szakorvos 10-20 év teljes szakmai gyakorlattal

Szakorvos több mint 20 év teljes szakmai gyakorlattal

**Munkavégzését az alábbiak közül melyik válasz jellemzi legjobban? (Egy válasz jelölhető!)**

Betegellátás, nagyrészt anesztézia

Betegellátás, nagyrészt intenzív terápia

Egyéb betegellátási feladatok

Oktatás

Adminisztráció

Egyéb nem betegellátáshoz köthető feladatok

**Fő munkahely (Egy válasz jelölhető!)**

Városi Kórház

Megyei Kórház

Egyetemi Klinika

Magánkórház

Egyéb

**Oktatási aktivitás (Egy válasz jelölhető!)**

Rendszeresen részt vesz szakorvosjelöltek oktatásában (átlagosan legalább heti 1 alkalommal)

Alkalmanként részt vesz szakorvosjelöltek oktatásában (átlagosan legalább havi 1 alkalommal)

Ritkábban oktat mint havi egy alkalom, de alkalomszerűen előfordul

Egyáltalán nem végez oktatói tevékenységet

**Elérhető-e a fő munkahelyének bármely munkaállomásán videolaringoszkóp? (Egy válasz jelölhető!)**

Igen

Nem

Nem tudom

**A fő munkahelyének mely munkaállomásain érhető el azonnal videolaringoszkóp? (Több válasz is jelölhető!)**

Nem érhető el egyáltalán videolaringoszkóp

Elérhető, de nem tudom hogy pontosan hol…

Sebészet

Szülészet

Fül-Orr-Gégészet

Szájsebészet

Mellkassebészet

Érsebészet

Urológia

Traumatológia

Sürgősségi Osztály

Ortopédia

Gyermeksebészet

Intenzív Osztály

Egyéb munkaállomás

**A fő munkahelyének mely alábbi munkaállomásain érhető el 10 percen belül videolaringoszkóp? (Több válasz is jelölhető!)**

Nem érhető el egyáltalán videolaringoszkóp

Elérhető, de nem tudom hogy pontosan hol…

Sebészet

Szülészet

Fül-Orr-Gégészet

Szájsebészet

Mellkassebészet

Érsebészet

Urológia

Traumatológia

Sürgősségi Osztály

Ortopédia

Gyermeksebészet

Intenzív Osztály

Egyéb munkaállomás

**Az alábbiak közül mely eszközről/eszközökről hallott már korábban? (Több válasz is jelölhető!)**

Airtraq (Prodol Meditec, Guecho, Spain)

AP Venner (Venner Medical GmbH, Danischenhagen, Germany)

Bonfils (Karl Storz, Slough, UK)

Bullard (Circon, ACMI, Stamford, CT, USA)

C-MAC (Karl Storz, Slough, UK)

C-MAC D-blade (Karl Storz, Slough, UK)

Coopdech (Daiken Medical, Osaka, Japan)

C-Trach (previously, Laryngeal mask company, Henley-on-Thames, UK)

GlideScope (Verathon UK, Amersham, UK)

King Vision VL (Ambu, St Ives, UK)

Levitan FPS (Clarus Medical, Minneapolis, MN, USA)

McGrath 5 (Aircraft Medical, Edinburgh, UK)

McGrath Mac (Aircraft Medical, Edinburgh, UK)

Pentax AWS (Pentax, Tokyo, Japan)

Shikani intubating stylet (Clarus Medical, Minneapolis, MN, USA)

Upsherscope (Mercury Medical, Clearwater, FL, USA)

Vividtrac (Vivid Medical, Palo Alto, USA)

Wuscope (Pentax Precision instruments, Orangeburg, NY, USA)

Egyéb videolaringoszkóp

Egyikről sem hallottam korábban

**Az alábbiak közül mely eszköz(ök) érhető(ek) el az Ön fő munkahelyén? (Több válasz is jelölhető!)**

Nem érhető el egyáltalán videolaringoszkóp

Elérhető de nem tudom, hogy milyen típus

Airtraq (Prodol Meditec, Guecho, Spain)

AP Venner (Venner Medical GmbH, Danischenhagen, Germany)

Bonfils (Karl Storz, Slough, UK)

Bullard (Circon, ACMI, Stamford, CT, USA)

C-MAC (Karl Storz, Slough, UK)

C-MAC D-blade (Karl Storz, Slough, UK)

Coopdech (Daiken Medical, Osaka, Japan)

C-Trach (previously, Laryngeal mask company, Henley-on-Thames, UK)

GlideScope (Verathon UK, Amersham, UK)

King Vision VL (Ambu, St Ives, UK)

Levitan FPS (Clarus Medical, Minneapolis, MN, USA)

McGrath 5 (Aircraft Medical, Edinburgh, UK)

McGrath Mac (Aircraft Medical, Edinburgh, UK)

Pentax AWS (Pentax, Tokyo, Japan)

Shikani intubating stylet (Clarus Medical, Minneapolis, MN, USA)

Upsherscope (Mercury Medical, Clearwater, FL, USA)

Vividtrac (Vivid Medical, Palo Alto, USA)

Wuscope (Pentax Precision instruments, Orangeburg, NY, USA)

Egyéb videolaringoszkóp

**Az alábbiak közül mely eszközt/eszközöket használta valaha a betegellátásban? (Több válasz is jelölhető!)**

Airtraq (Prodol Meditec, Guecho, Spain)

AP Venner (Venner Medical GmbH, Danischenhagen, Germany)

Bonfils (Karl Storz, Slough, UK)

Bullard (Circon, ACMI, Stamford, CT, USA)

C-MAC (Karl Storz, Slough, UK)

C-MAC D-blade (Karl Storz, Slough, UK)

Coopdech (Daiken Medical, Osaka, Japan)

C-Trach (previously, Laryngeal mask company, Henley-on-Thames, UK)

GlideScope (Verathon UK, Amersham, UK)

King Vision VL (Ambu, St Ives, UK)

Levitan FPS (Clarus Medical, Minneapolis, MN, USA)

McGrath 5 (Aircraft Medical, Edinburgh, UK)

McGrath Mac (Aircraft Medical, Edinburgh, UK)

Pentax AWS (Pentax, Tokyo, Japan)

Shikani intubating stylet (Clarus Medical, Minneapolis, MN, USA)

Upsherscope (Mercury Medical, Clearwater, FL, USA)

Vividtrac (Vivid Medical, Palo Alto, USA)

Wuscope (Pentax Precision instruments, Orangeburg, NY, USA)

Egyéb videolaringoszkóp

Egyiket sem

**Milyen gyakran használ videolaringoszkópot? (Egy válasz jelölhető!)**

Soha

Naponta

Hetente

Havonta

Évente

Ritkábban mint évente

**Milyen cél(ok)ból használ videolaringoszkópot? (Több válasz is jelölhető!)**

Nem használok egyáltalán

„Rutin” légútbiztosítás

"Előre látható nehéz légút" megoldása

"Váratlan nehéz légút" megoldása

Oktatás

Egyéb

**Milyen oktatásban részesült a videolaringoszkópiát illetően? (Egy válasz jelölhető!)**

Nem kaptam oktatást és nem is használok ilyen eszközt

Nem kaptam oktatást de használok videolaringoszkópot

Kötelező oktatás szimulátoron

Kötelező oktatás betegeken

Önkéntes alapú oktatás szimulátoron

Önkéntes alapú oktatás betegeken

**Mit tekint a videolaringoszkópia klinikai alternatívájának? (Több válasz is jelölhető!)**

Direkt laringoszkópiát

Fiberoszkópot

Laringeális maszkot

Sebészi légútat

Egyéb eszközt

Nem ismerek alternatív eszközt/eljárást

**Mi alapján választották ki az Ön fő munkahelyén azt, hogy mely típusú eszköz legyen elérhető? (Több válasz is jelölhető!)**

Nincs videolaringoszkóp a fő munkahelyemen

Nem tudom, hogy mi alapján választották ki a meglévő eszközt

Az eszköz beszerzési költsége

Rövid klinikai kipróbálás során szerzett tapasztalatok

Korábbi tudományos publikációkban leírt tapasztalatok

Munkahelyi vezető döntése

Légútbizosításban jártas és aziránt érdeklődő kolléga véleménye alapján

Szakmai vélemény nélküli eszközbeszerzés

**Mi a véleménye a videolaringoszkópokról? (Egy válasz jelölhető!)**

Nincs velük érdemi tapasztalatom, így megítélni sem tudom

Haszontalan "kütyük", érdemi klinikai előnyük nincs

Hasznos eszközök, minden légútbiztosítás esetében ezt kellene használni

Hasznos eszközök, de érdemi előnyük csak bizonyos helyzetekben érvényesül igazán

**Milyen légútbiztosítási probléma/problémák megoldására használható Ön szerint eredményesen videolaringoszkóp? (Több válasz is jelölhető!)**

Szájnyitási képtelenség

Hangrés feltárásának/látótérbe hozásának nehezítettsége

Tubusbevezetési nehézség

Váladékkal/vérrel "szennyezett" légút

Gégeödéma

Igazolt vagy feltételezett traumás nyaki gerincszakasz sérülés

Egyikre sem a fentiek közül
